# What contributes to a good quality of life in early dementia? awareness and the QoL-AD: a cross-sectional study

**DOI:** 10.1186/1477-7525-12-94

**Published:** 2014-06-11

**Authors:** Robert T Woods, Sharon M Nelis, Anthony Martyr, Judith Roberts, Christopher J Whitaker, Ivana Markova, Ilona Roth, Robin Morris, Linda Clare

**Affiliations:** 1 DSDC Wales, Bangor University, Ardudwy, Holyhead Road, Bangor, UK; 2 School of Psychology, Bangor University, Brigantia Building, Penrallt, Bangor, UK; 3 NWORTH, Bangor University, Y Wern, Holyhead Road, Bangor, UK; 4 Department of Psychiatry, University of Hull, Hull, UK; 5 Department of Life Sciences, Open University, Walton Hall, Milton Keynes, UK; 6 Department of Psychology, Institute of Psychiatry, Kings College London, De Crespigny Park, London, UK

**Keywords:** Quality of life, Anosognosia, Insight, Self‒awareness, Meta‒cognition, Alzheimer’s disease, Vascular dementia, Self-concept, Conscientiousness

## Abstract

**Background:**

Self-report quality of life (QoL) measures for people with dementia are widely used as outcome measures in trials of dementia care interventions. Depressed mood, relationship quality and neuropsychiatric symptoms predict scores on these measures, whereas cognitive impairment and functional abilities typically do not. This study examines whether these self-reports are influenced by personality and by the person’s awareness of his/her impairments. A strong negative association between QoL and awareness of deficits would have implications for the validity of self-report in this context and for therapeutic interventions aiming to increase adjustment and coping.

**Methods:**

Participants were 101 individuals with early‒stage dementia and their family carers participating in the Memory Impairment and Dementia Awareness (MIDAS) Study. QoL was assessed using the QoL-AD scale, and awareness was assessed in relation to memory, activities of daily living and social functioning. Self-concept, conscientiousness, quality of relationship and mood were assessed and a brief neuropsychological battery administered. Carers rated their own stress and well-being and reported on neuropsychiatric symptoms. A series of regression analyses predicting QoL-AD were carried out, identifying key variables in each domain of assessment to take forward to an overall model.

**Results:**

Cognitive impairment was not related to QoL. The final model accounted for 57% of the variance in QoL-AD scores, with significant contributions from depressed mood, severity of irritability shown by the person with dementia, self-concept, quality of relationship (rated by the person with dementia) and male gender. The bivariate relationships of QoL-AD with awareness of memory function, awareness of functional abilities and conscientiousness were mediated by both depressed mood and self-concept.

**Conclusions:**

This study reports the most comprehensive approach to evaluation of awareness to date. Most of the indices of awareness used are not related to self-reported QoL. Discrepancies in evaluative judgements of memory function and functional abilities between people with dementia and carers are related to QoL, but this relationship is mediated by both depressed mood and self-concept, which have a much stronger relationship with QoL. The validity of self-report measures of QoL in people with early stage dementia is supported by these results.

## Background

The evaluation of outcomes for people with mild to moderate dementia has been transformed in recent years by the development of tailored self-report disease-specific quality of life (QoL) measures [1]. Where previously cognitive function, functional abilities, nursing home placement and proxy measures of quality of life and well-being were the only available outcome options, a number of measures have now been developed that people with dementia are able to complete reliably and consistently, enabling the perspective of the person with dementia to be heard first-hand.

Considering findings from their systematic review, Banerjee et al. [1] conclude that more research is needed on the factors associated with and contributing to lower and greater quality of life in people with dementia. Such research can inform the continuing debate on the validity and meaningfulness of the self-report of quality of life by people with dementia. This would more fully address concerns that memory and other cognitive impairments as well as a lack of awareness of deficits potentially influence the ‘accuracy’ of reporting [2,3].

The range of available disease-specific measures continues to increase, each with particular emphases and applicability, all typically showing moderate inter-correlations with each other. This paper focuses on the QoL-AD [4,5], a brief 13-item scale administered in an interview with the person with dementia. A review of outcome measures suggested that it was the measure of choice in the evaluation of quality of life in psychosocial interventions [6]. A proxy version is available, but here we will focus on self-report, as the person’s own perspective is fundamental to quality of life assessment.

A number of studies have reported on a range of variables potentially associated with QoL-AD scores. Some of these studies involved people with moderate to severe dementia. The results of these studies are tabulated in Additional file 1. They mirror those reported by Banerjee et al. [1] across the range of variables that have been explored. Depressed mood is the most common predictor of low quality of life; cognitive function (as measured by the MMSE, for example) consistently shows no association with quality of life [7,8]. Other variables identified as associated with higher QoL in some studies include having a spouse caregiver and not having a negative relationship with the caregiver [9] and having fewer neuropsychiatric problems [10-12]. Functional abilities are not related to quality of life in most of the studies included. It is clear that there is scope for identifying other factors contributing to the prediction of quality of life in people with dementia, with a fair amount of variance remaining to be explained, with the largest study accounting for only 42% of the variance [11].

Amongst the potential influences on quality of life that have yet to be examined fully in relation to the QoL-AD are aspects of personality and the person’s awareness of abilities and deficits. Self-concept, which comprises the person’s self-knowledge across a number of domains, including personality traits and attributes, has recently been shown to predict QoL in people with dementia, independent of its relationship with anxiety and depression [13]. Specific personality traits are likely to be a strong influence on QoL throughout the lifespan, but do not yet appear to have been examined specifically in the context of QoL in dementia [1]. Of the ‘Big 5′ personality variables, conscientiousness emerges as of particular importance both in relation to risk of developing dementia and in personality change in dementia. People with high levels of conscientiousness have been reported to have a reduced risk of developing dementia [14] and it is the trait of conscientiousness which is reported to show most change (a reduction) in people who have been diagnosed as having a dementia [15]. It is possible that people with dementia who have high levels of conscientiousness may be especially sensitive to the development of cognitive deficits, which make it difficult for them to maintain the high standards they set for themselves. This would contribute to lower QoL in these individuals, unless such individuals show lower awareness of deficits, through a defensive denial mechanism [16,17]. The relationship between conscientiousness and awareness of deficits is uncertain, with a recent study [18] reporting that higher levels of conscientiousness were related to *greater* awareness of memory difficulties, in contrast to an earlier report [16]. Clearly, it would be helpful to consider both conscientiousness and awareness of deficits together in considering the influence of each on QoL.

Awareness can be defined as ‘a reasonable or realistic perception or appraisal of a given aspect of one’s situation, functioning or performance, or of the resulting implications’ [19]. This broad definition encompasses different awareness phenomena which can be regarded as operating at different levels and which are elicited and assessed in different ways and which require consideration of, and evaluation in relation to, specific objects. Four levels of awareness have been delineated which reflect increasingly broad aspects of the definition [20], and which lend themselves to different approaches to assessment, from behavioural observation to in-depth interview:

• sensory registration - the most basic level of awareness of the immediate environment.

• performance monitoring - the person’s ability to monitor his/her performance on a specific task (e.g. a memory test).

• evaluative judgement - the person’s evaluation of his/her ability and function in a specific domain (e.g. memory).

• meta-representation - the person’s reflection on his/her situation and any changes e.g. relating to a health condition (in this case, dementia), its effects and its nature.

Each level of awareness needs to be evaluated in relation to a specified object of awareness, in that a person’s awareness may vary according to the particular object, whether it be memory, functional ability or knowledge of the diagnosis. The different indices and measures of awareness fall into two main types [21]. Firstly, general ratings of awareness based on interviews conducted by a clinician or researcher, are often described as measures of ‘insight’. Secondly, indices based on discrepancies have been used. These may compare self-ratings made by the person with dementia with those made by an informant (often a family care-giver) on parallel questionnaires, or calculate discrepancies between the person’s estimate of his/her performance on a task or test and the actual task performance. All have some drawbacks, and the case has been made for multi-dimensional evaluation [20].

One study [22] has examined QoL-AD scores in relation to an assessment of awareness. A global judgement was made on a 3 point scale after a neuropsychological assessment and an interview with the patient (N = 48) and carer. It is difficult to identify the specific object of awareness assessed, or the specific level (as elements of both meta-representation and evaluative judgement appear to have been included). No significant differences in QoL-AD scores were found between the different categories, although those with full awareness had lower scores than those with no awareness. Lack of awareness did contribute to the discrepancy between proxy- and self-ratings on the QoL-AD.

Other studies examining the relationship between QoL and awareness have used alternative QoL measures. Two simple dichotomous indices of awareness, a researcher-rating of presence or absence of ‘insight’ and the person’s response to the question ‘do you have problems with memory or thinking?’ (an evaluative judgement) were correlated with self-ratings on the DEMQOL instrument [23] in a much larger sample (174 people with mild to moderate Alzheimer’s) [24]. QoL self-ratings were overall higher in those with impaired awareness, although this effect was only significant in relation to a sub-group with moderate dementia, not for those with milder cognitive impairment.

The Memory Awareness Rating Scale - Memory Functioning Scale (MARS-MFS), focusing on evaluative judgements of memory function has been used in one study [25]. Scores on this scale are used to calculate the discrepancy between the person’s and a carer’s ratings of the person’s memory function [26]. The Bath Assessment of Subjective Quality of Life in Dementia (BASQID) was used to evaluate QoL in a sample of 69 people with dementia. Those with lower awareness (i.e. who rated their memory function much more positively than their carer) also gave higher self-reports of QoL. A model comprising enjoyment of activities, activity of daily living function and the MARS-MFS discrepancy score predicted 40% of the variance of self-reported QoL scores, with memory awareness being the strongest predictor.

The current study aims to add to our understanding of the influences on self-reported QoL in mild dementia by examining a broader range of influences than that included in previous studies of the QoL-AD. Specifically, the influence of awareness, measured rigorously across several domains, and conscientiousness will be evaluated alongside variables already known to be important, including depression, self-concept, neuropsychiatric symptoms, aspects of the care-giving situation, and relationship quality. If self-reports of QoL do have validity and are meaningful, it is predicted that awareness will be only one of a number of sources of influence, and that cognitive impairment will not be related to QoL in mild dementia. The suggestion [24] “that impairment of insight may actually be protective for the person with dementia” (p. 334), in the sense of maintaining QoL, will be evaluated using a more rigorous approach to the evaluation of awareness.

## Methods

The data presented here are drawn from the first wave of the Memory Impairment and Dementia Awareness Study (MIDAS), comprising 101 people in the early stages of dementia recruited from NHS Memory Clinics in North Wales, UK. Ethical approval for the study was granted by the relevant University and NHS Ethics Committees.

### Participants

Inclusion criteria: an ICD-10 diagnosis of Alzheimer’s disease (AD), vascular dementia or mixed Alzheimer’s and vascular dementia [27]; a score of 18 or above on the Mini-Mental State Examination (MMSE [28]); ability to communicate verbally in English, and availability of a spouse, partner, other family member or friend (‘carer’). Exclusion criteria: concurrent major depression, psychosis or other neurological disorder; and past history of neurological disorder, stroke or brain injury. All people with dementia from the Memory Clinics who were identified as potentially eligible from screening of clinical notes were invited to consider joining the study by members of the clinical team, either in person or by letter where a clinic visit was not pending. Participants were seen at home (unless they preferred to be seen at the University) and people with dementia and carers were seen separately. All gave informed consent. Two to three visits of up to two hours duration were typically required.

### Measures

Quality of Life was assessed using the QoL-AD [4], a simple self-report measure of quality of life, with 13 items, each rated by the person on a 4 point scale, in the course of an interview. The items include Energy; Fun; Money; Physical health; Friends; Family; etc. The measure shows good internal consistency and good re-test reliability over a period of one week [29]. Higher scores indicate better quality of life.

The mood of the person with dementia was assessed with the Hospital Anxiety and Depression Scale (HADS [30]); higher scores indicate greater levels of anxiety and depression respectively. Self-concept was evaluated with the Tennessee Self-Concept Scale, Short Form (TSCS [31]), and the personality trait of conscientiousness was assessed with the NEO-Five Factor Inventory conscientiousness sub-scale (NEO-FFI-C [32]. Higher scores indicate stronger self-concept and greater conscientiousness respectively. These scales were selected as relatively brief, established indicators of the domains in question in the general population, that have all been used and found suitable in previous studies with people with early dementia [16,33]. The person with dementia also rated the quality of his/her relationship with the care-giver, using the 5-item Positive Affect Index [34], with higher scores reflecting a more positive relationship.

A brief neuropsychological battery was used to assess the cognitive function of the person with dementia. Cognitive domains assessed included: premorbid IQ (National Adult Reading Test; NART [35]); everyday memory function: (Rivermead Behavioural Memory Test, total score; RBMT [36]); episodic memory (immediate recall score, Wechsler Memory Scale, Word List subtest [37]); semantic knowledge (Pyramids & Palm Trees, picture–picture matching [38]); language (Graded Naming Test [39]); and verbal fluency as an index of executive function (Delis–Kaplan Executive Function System: D-KEFS, letter and category fluency [40]). For all the cognitive measures, higher scores indicate better function, with the exception of the NART error score, where fewer errors indicate higher estimated premorbid IQ.

Carers completed proxy versions of the TSCS, and the NEO-FFI conscientiousness sub-scale, providing their perspective on the self-concept and conscientiousness of the person with dementia. They also rated the occurrence and severity of neuropsychiatric symptoms shown by the person with dementia (including depression, apathy, hallucinations etc.) using the Neuropsychiatric Inventory Questionnaire (NPI-Q [41]). Higher scores indicate the occurrence of more symptoms occurring or greater severity. They additionally rated their own level of distress in relation to their own reaction to these symptoms, as well as completing questionnaires on their stress related to care-giving (the Relatives Stress Scale, RSS [42]) and more general psychological well-being (General Health Questionnaire, 12 item version, GHQ-12 [43]). Higher NPI-Q distress scores, and higher scores on the RSS and GHQ are all associated with greater difficulty experienced by carers. Carers also rated the relationship, from their own perspective, with the person with dementia, again using the Positive Affect Index, with higher scores indicating a more positive relationship.

The measures of awareness used in this study have been fully described elsewhere [44]. The four objects of awareness included were: memory, instrumental activities of daily living (IADL), social behaviour and metacognitive reflection on the condition, its implications and impact. For memory, IADL and social behaviour, awareness at the level of evaluative judgement was assessed using discrepancies between the ratings of the person’s function made by the person with dementia and by the carer on an appropriate measure; for memory, the Memory Awareness Rating Scale Memory Functioning Scale (MARS-MFS [26]); for IADL, a slightly amended Functional Activities Questionnaire (FAQ [45,46]); for social behaviour, including emotional recognition and empathy, the Socio-Emotional Questionnaire (SEQ [47,48]). In each case, the discrepancy between the two scores was divided by the mean, to correct for scaling effects [49]. This yielded memory functioning discrepancy (MFD), functional activity discrepancy (FAD) and social functioning discrepancy (SFD) scores. Discrepancy scores close to zero indicate good agreement between the person with dementia and the carer. Positive scores indicate that self‒rating is higher than the carer rating, and vice versa. Higher scores may then be said to indicate a lower level of awareness.

For memory, an assessment of awareness at the level of performance monitoring was also undertaken, using the Memory Awareness Rating Scale Memory Performance Scale (MARS-MPS: [26]) which compares the person’s rating of performance on the sub-tests of the Rivermead Behavioural Memory Test, immediately after being carried out, with actual performance. Here the index of awareness is the ratio of the ratings to the actual score (the Memory Performance Ratio, MPR). Again, discrepancies close to zero reflect good agreement, and higher scores increasing divergence between actual and self-rated performance, indicating lower awareness.

Finally, as an index of the metacognitive reflective level of awareness, Global Interview Ratings were used. Interviews lasting between 10 and 60 minutes were conducted independently with the person with dementia and carer, following an interview guide which aimed to explore the person’s understanding of his/her condition and situation. Transcripts were then rated by members of the research team on a 5 point scale (5 = extensive evidence of awareness; 1 = no evidence of awareness), based on both interviews from the dyad. Inter-rater reliability was assessed on a sample of transcripts and found to be satisfactory. The percentage agreement for the global awareness ratings was 88.9% agreement (N = 18; 16 agreements, 2 disagreements of one scale point; Cohen’s Kappa = .85 [44]). Higher scores here indicate greater awareness.

### Data analysis

Correlational analyses explored the bivariate association between quality of life and the other variables, and their size interpreted according to Cohen’s criteria [50] (0.1 small; 0.3 medium; 0.5 large). In order to develop a predictive model for QoL-AD scores, a multiple regression analysis was planned. To reduce the number of variables to be entered into one overall regression analysis to produce the final model predicting QoL-AD scores, the following method was adopted, which maximised the potential for a strong prediction by selecting the most predictive variables from each domain, and allowed identification of the domains which appeared most relevant to QoL-AD scores. Accordingly, six separate regression analyses were conducted, one for each domain of variables. Initially a backward regression analysis on the person with dementia background variables (including MMSE score of the person with dementia), was performed. Then age and gender and any other variables reaching significance at the 5% level in this analysis were included in each of the subsequent five backward regression analyses with the respective variables from that domain. These five domains were: person with dementia – psychosocial (self-ratings); person with dementia – neuropsychological; caregiver ratings of the person with dementia; caregiver background and self-ratings; and the awareness measures. The backward regression analyses were performed in SPSS v.15 and used the default criterion probability of F-to-remove > = .10. For the NPI-Q variables, regression analyses were run on the symptoms showing significant bivariate correlations with QoL-AD, for severity and distress separately. Symptoms showing a significant relationship with the QoL-AD in these multivariate analyses were then included in analyses relating to ‘caregiver ratings of the person with dementia’ (severity) and ‘caregiver self-ratings’ (distress). Variables reaching significance in the analyses in the five domains at the 5% level were taken forward into the final analysis, with age and gender. For the final analysis, the model selected was that accounting for the greatest variance (adjusted R^2^). No problems with collinearity were identified.

## Results

The participants were 101 individuals (54 female, 47 male) with early-stage dementia. The participants’ characteristics have been described in detail in previous reports on this cohort [18]. Briefly, the mean age of participants with dementia was 78.7 years (sd 7.7, range 51–91), with half (51) having a diagnosis of Alzheimer’s, 30 vascular dementia and 20 mixed Alzheimer’s and vascular dementia. Mean MMSE score was 24.2 (sd 2.8; range 18–30) and 57 were receiving acetylcholinesterase-inhibiting medication. The 101 carers (64 female, 37 male) had a mean age of 68.4 years (sd 14.0, range 33–89). Two-thirds (66) were spouses/partners of the person with dementia, 26 were adult children and the remaining nine were evenly split between siblings, nieces/nephews and friends. All participants were white European, representing the demographics of the study area. Five participants completed only part of the assessment. In a few cases, individual participants were unable to complete one or more of the tasks, for reasons such as physical or sensory impairment, or difficulty understanding task demands.

Bivariate correlations (Pearson’s) with the person’s rating of quality of life (QoL-AD) indicated a number of statistically significant correlations. These are shown in Table 1, where it is clear that these occur across the six domains considered, with the exception of the group of variables reflecting the background characteristics of the person with dementia. Large correlations are with other scales completed by the person with dementia, notably the depression scale from the HADS, self-concept and the rating by the person with dementia of the quality of relationship with his/her relative. Conscientiousness shows a medium positive, significant bivariate correlation with QoL-AD (r = 0.29). The informant ratings of the self-concept of the person with dementia and of the quality of relationship generally mirror the associations evident in the ratings made by the person with dementia, albeit at a lower level. However, the informant’s rating of the trait of conscientiousness shown by the person with dementia did not show an association with QoL-AD (r = -0.09). Whilst the correlation of QoL-AD with MMSE scores is close to zero, two of the neuropsychological tests show a small to medium correlation with QoL-AD (total RBMT score and category fluency from the D-KEFS). Awareness scores in the areas of memory function and functional abilities show a small to medium correlation with QoL-AD, but the Global Interview Ratings of awareness and awareness scores related to socio-emotional functioning and memory performance are not significantly related to QoL-AD in this sample.

**Table 1:** Bivariate correlation between QoL-AD and other variables in six domains

**Domain**	**QoL-AD (self-report)**	**Domain**	**QoL-AD (self-report)**
**Person with dementia background**		**Person with dementia psychosocial (self-ratings)**	
Age	0.16	HADS anxiety	-0.45**
MMSE score	0.02	HADS depression	-0.61**
Years of education	-0.03	NEO-FFI conscientiousness	0.29**
Socio-economic status	-0.08	TSCS self-concept	0.52**
		Quality of relationship	0.50**
**Person with dementia neuropsychological**		**Awareness indices**	
NART errors	-0.10	MFD	0.21*
WMS-III Word List total recall	0.16	FAD	0.22*
RBMT Total score	-0.21*	SFD	0.11
GNT raw score	-0.04	MPR	0.16
Pyramids & Palm Trees raw	0.15	Global Interview Rating	0.06
D-KEFS letter fluency total	0.16		
D-KEFS category fluency total	0.22*		
**Caregiver ratings of person with dementia**		**Caregiver background and self-ratings**	
NPI-Q symptom score	-0.30**	Age	0.02
NPI-Q severity score	-0.33**	Socio-economic status	-0.11
NEO-FFI conscientiousness	-0.09	NPI-Q distress score	-0.33**
TSCS self-concept	0.40**	GHQ-12	-0.11
		Relatives Stress Scale	-0.24*
		Quality of relationship with PwD	0.22*

* p < 0.05; **p < 0.01.

Significance levels are given as indicative only, in view of multiple comparisons, and are included to provide a full presentation of the data.

*Key to abbreviations: HADS Hospital Anxiety and Depression Scale, TSCS Tennessee Self-Concept Scale, NEO-FFI NEO Five-Factor Inventory, NART National Adult Reading Test, WMS-III Wechsler Memory Scale, 3*^*rd*^*edition, D-KEFS Delis-Kaplan Executive Function System, NPI-Q Neuropsychiatric Inventory Questionnaire, GHQ-12 General Health Questionnaire.*

All three NPI-Q indices – reflecting both the severity of the symptoms and their effect on the carer - are associated with reduced quality of life, as is the degree of stress reported by the carer. Table 2 shows the profile of occurrence of the 12 symptoms included in the NPI-Q, together with the correlations of each of the NPI-Q dimensions with QoL-AD. Anxiety is the symptom most commonly reported as having occurred in the past month (in 60% of participants), with apathy (58%), depression (54%), and irritability (51%) all occurring in at least half the sample. The severity of each of these 4 symptoms was significantly negatively correlated with QoL-AD scores, together with agitation/aggression, sleep problems and difficulties with appetite and eating. Carers’ highest ratings of distress arising directly from the behaviour of the person with dementia were reported in relation to the presence of delusions, agitation, depression, irritability, anxiety and hallucinations. The carer’s distress related to irritability, agitation/aggression, appetite/eating problems, sleep problems, apathy and depression showed significant correlations with the QoL-AD scores reported by the person with dementia.

**Table 2:** NPI-Q symptoms and correlation with QoL-AD

**NPI-Q symptom**	**Occurrence**^**1**^	**Occurrence & QoL-AD correlation**	**Severity & QoL-AD correlation**	**Distress & QoL-AD correlation**	**Mean distress score when symptom is present (range 1–5)**
	**N = 100**				
Delusions	13	-.05	-.03	-.03	3.38
Hallucinations	10	-.16	-.13	-.13	2.20
Agitation/aggression	44	-.25*	-.25*	-.30**	2.59
Depression	54	-.18	-.20*	-.23*	2.48
Anxiety	60	-.16	-.22*	-.07	2.28
Elation	12	-.04	-.10	-.10	1.50
Apathy/indifference	58	-.24*	-.21*	-.24*	1.83
Disinhibition	19	-.09	-.11	-.15	2.0
Irritability	51	-.26**	-.35**	-.31**	2.29
Motor disturbance	28	.20	.09	.09	1.82
Night time/sleep	45	-.17	-.25*	-.26*	1.47
Appetite/eating	47	-.22*	-.24*	-.30**	2.04
Total	Mean 4.41 (SD 2.52)	-.30**	-.33**	-.33**	2.14

^1^Yes/no in past month.

* p < 0.05; **p < 0.01.

The variables meeting the criteria to be included in the final regression analysis were as follows (see Table 3):

**Table 3:** Results of regression analyses in each of 6 domains

	**Adjusted R2**		**Standardised β**	**Significance**
**Person with dementia – background**	0.10	F = 4.589, p = 0.005		
Age			.252	.014
Gender			-.169	.094
Alzheimer’s v mixed			.322	.002
**Person with dementia – psychosocial (self-rated)**	0.52	F = 21.39, p < 0.0001		
HADS - depression			-.403	<.0001
TSCS self-concept			.203	.021
Quality of relationship			.227	.007
**Person with dementia - neuropsychological**	0.19	F = 4.905, p = 0.001		
Category fluency			.269	.016
**Caregiver ratings of person with dementia**	0.23	F = 6.727, p <0.0001		
TSCS self-concept (informant)			.227	.035
NPI-Q irritability severity			-.221	.036
**Caregiver self-ratings and background variables**	0.18	F = 5.95, p < 0.0001		
NPI-Q agitation/aggression distress			-.310	.002
**Awareness indices**	0.16	F = 5.306, p = 0.001		
FAD			.217	.037

Results of preliminary regression analyses in six domains, showing only variables selected for inclusion in final model. Excluded variables are listed in the text.

Background variables: diagnosis (Alzheimer’s v vascular/mixed); *not* MMSE score, education or socio-economic status.

Psychosocial factors rated by the person with dementia: HADS-depression, TSCS self-concept and quality of relationship; *not* NEO-FFI conscientiousness or HADS-anxiety.

Neuropsychological test scores: only D-KEFS category fluency; *not* RBMT, NART errors, WMS-III word list total recall, Graded Naming Test score, Pyramids & Palm Tree semantic knowledge score or D-KEFS letter fluency.

Carer’s ratings of the person with dementia: informant rating of TSCS self-concept and symptom severity rating for NPI-Q-irritability; *not* NEO-FFI conscientiousness. NPI-Q symptom severity ratings for agitation/aggression, depression, anxiety, apathy, sleep problems, eating/appetite problems were excluded in the preliminary NPI analysis.

Carer’s own ratings and background factors: Distress rating NPI-Q-agitation/aggression; *not* carer’s age, socio-economic status, rating of quality of relationship, GHQ-12 or Relatives Stress Scale score. Distress ratings on NPI-Q-agitation/aggression, NPI-Q-apathy, NPI-Q-depression and NPI-Q-sleep problems were excluded in the preliminary NPI-Q analysis.

Awareness indices: FAD; *not* Global Interview Ratings or MPR or MFD or SFD.

Thus these analyses reduced the number of variables included in the final analysis from 43 to 11.

The regression model obtained in the final analysis (see Table 4) explained 57% of the variance in QoL-AD scores and comprised eight of the eleven variables entered. Variables removed from the model were: Informant rated self-concept, NPI-Q-distress relating to agitation/aggression and age. Self-rated depressed mood and NPI-Q-severity related to irritability were the most influential predictors of reduced quality of life. Positive self-concept and a good quality of relationship were related to increased quality of life, as was male gender. Higher scores on the category fluency test and a positive self-evaluation of functional ability also contributed to the prediction of higher quality of life, although these beta coefficients were of borderline statistical significance. Having a diagnosis of Alzheimer’s also remained in the model with the greatest predictive power, with a non-significant beta coefficient.

**Table 4:** Final regression analysis – predictors of QoL-AD

	**QoL-AD**	
	**Standardised β**	**Significance**
**Person with dementia - background**		
Age	-	
Gender	-.154	.035
Alzheimer’s v mixed	.124	.106
**Person with dementia – psychosocial (self-rated)**		
HADS depression	-.332	.0001
TSCS self-concept	.218	.007
Quality of relationship	.195	.019
**Person with dementia – neuropsychological**		
D-KEFS Category Fluency	.139	.055
**Carer ratings of person with dementia**		
NPI-Q irritability severity	-.281	.0001
**Caregiver self-ratings and background variables**		
NPI-Q agitation/aggression distress	-	
TSCS self-concept informant rating	-	
**Awareness indices**		
FAD – functional activities discrepancy	.151	.051

Adjusted R^2^ = 0.573; F 12.01, p < 0.0001.

Given that both FAD and MFD had significant bivariate associations with QoL-AD, we explored whether this relationship could be mediated by the major predictors, depression and self-concept. In relation to depression, FAD and MFD do have significant negative correlations with scores on the HADS depression scale (-0.24 and -0.41 respectively). The significant relationship between MFD and QoL-AD (β = 0.263; p = 0.009), is lost when depression score is added to the model (β = -.0.074; p = 0.441). Similarly, removing depression from our final model would result in the contribution of the index of awareness (FAD) (β = 0.202; p = 0.017) being significant. This means that in our data the contribution of FAD and MFD to QoL is indeed mediated by mood, meeting established criteria for mediation [51].

In relation to self-concept, the bivariate correlations with FAD and MFD are 0.26 (p = 0.011) and 0.47 (p < 0.001) respectively. Self-concept clearly mediates the relationship between MFD and QoL-AD, with the relationship between these variables losing statistical significance when self-concept is added to the model (β changes from 0.263, p = 0.009, to -0.028; p = 0.803). The mediation effect is less marked for FAD, when tested in the main regression model (retaining all other variables), with only a small change in standardised beta when self-concept is removed (β changes from 0.151, p = 0.051, to 0.162; p = 0.049). However, in a simpler model, with only FAD and the demographic variables, beta changes from 0.206 (p = 0.045) to 0.125 (p = 0.173) when self-concept is added.

Similar mediation effects are evident for conscientiousness, which has bivariate correlations of -0.22 (p = 0.03) with depression and 0.43 with self-concept (p < 0.001). Standardised beta for conscientiousness changes from 0.237 (p = 0.015) to 0.123 (p = 0.123) when depression is added to the simple model (including demographic variables only) predicting QoL-AD and to 0.074 (p = 0.44) when instead self-concept is added.

## Discussion

The present study adds to our knowledge of the variety of factors that contribute to QoL judgements made by people in the early stages of dementia. The 57% of the variance (adjusted R^2^) accounted for by the current model exceeds previous findings for the self-report QoL-AD, which have ranged from 13% to 42% (see Additional file 1). Before considering the variables included in the final model, it is important to emphasise that neither conscientiousness, as rated by the person with dementia, nor four of the five awareness measures emerged as significant predictors in the multivariate analyses. Conscientiousness and the discrepancy between carer and person with dementia evaluations of memory did show small to medium, significant bivariate correlations with QoL-AD. We have shown that the association of these variables with QoL is mediated by depression and self-concept in constructing the final model.

Our results at first sight appear discrepant from those reported by Trigg and colleagues [25], where a similar memory awareness measure was the strongest single predictor of QoL (albeit measured with a different instrument). It should be noted that their study used a simple discrepancy score, and not the corrected discrepancy score we have developed [49]. The bivariate correlation they report (0.31) is a little higher than in our study (0.21), but still relatively small. A key difference is that they do not include a self-report measure of depression in their model, despite this consistently being the strongest predictor across a number of studies. If we had not included depression in our model, as we have shown in the mediation analyses, awareness would have featured more prominently, but the proportion of variance explained would fall to 50.7%.

Whilst our findings do not support a primary role for conscientiousness and awareness in predicting QoL, they do bring together a number of consistent predictors, and confirm their independent effects, resulting in a much fuller account of the influences on QoL in dementia than has been possible previously. Thus we see self-concept making a strong independent contribution alongside a range of other variables, including depressed mood. A more positive view of self (on the Tennessee Self-Concept Scale) is associated with higher QoL-AD scores. We have previously shown that a more positive self-concept is related to greater discrepancies in reports of memory and social functioning between people with dementia and their carers [13], and the role of self-concept in mediating the relationship between some aspects of awareness and QoL-AD is clear from the current study. Informant ratings of self-concept showed bivariate correlation with QoL-AD, but did not add to the final model.

Entirely consistent with the previous literature, low depression scores emerge as the strongest predictor of self-reported QoL. Consistent also with previous reports [9] the quality of the relationship with the caregiver, as rated by the person with dementia, also adds to the prediction of self-reported QoL. Even in early dementia, relationships with carers become a pivotal component of the experience of living with the condition [52]. Just as the quality of the relationship has a major influence on the well-being of the carer [53], so it also has a similarly important influence on the person with dementia. The importance of this domain is reflected in several items on relationships contributing to the QoL-AD total score.

Consistent with previous reports, general cognitive impairment, as assessed by the MMSE, showed no association with the QoL-AD in this population. A specific aspect of cognitive function – category fluency – did show a positive association with QoL ratings, however. This could reflect an association with this aspect of executive function, but the relationship with letter fluency was not significant. Although it is possible that low scores on category fluency are associated with word finding difficulties which interfere with communication and interaction more obviously than do memory difficulties per se, and impact negatively on QoL for this reason, the lack of association between QoL and Graded Naming Test scores does not support this explanation. Conversely, performance on a memory test shows a small to medium negative association, in bivariate correlations, with QoL-AD, suggesting that those with better memory performance are reporting lower quality of life.

Neuropsychiatric symptoms were relatively common in this sample of people with early dementia, with anxiety, depression, irritability and apathy being commonly reported. Severity of irritability symptoms made a strong contribution to the final model, with higher quality of life associated with lower irritability reported by the caregiver. A number of mood symptoms were related to quality of life in bivariate correlations, and the specific symptom entering the model may be seen as representing a spectrum of low mood and distress. The contribution of neuropsychiatric symptoms to QoL-AD scores is consistent with some previous studies (e.g. [11]), with the mood symptoms being prominent in some (e.g. [10]).

Caregiver distress, both in relation to specific NPI-Q symptoms and more generally on the Relatives Stress Scale, was also associated with lower QoL as rated by the person with dementia, suggesting a sensitivity on the part of the person with dementia to the emotional climate, as has been demonstrated previously [54]. The carer’s NPI-Q distress rating relating to agitation/aggression did not however contribute additionally to the final model.

Demographic variables, including age, gender, socio-economic status and years of education, were not associated with QoL-AD ratings in bivariate correlations, but gender (being male) and diagnosis (Alzheimer’s vs. vascular or mixed dementia) did contribute to the final model, with the beta coefficient for gender being statistically significant.

In the area of the relationship between QoL and awareness, our findings add to the few previous attempts to address this area. Our results indicate that two areas of awareness – evaluative judgements of memory function and evaluative judgements of functional abilities, based on discrepancies between the reports of the person with dementia and those of a caregiver, show small to medium bivariate correlations with self-reported QoL-AD, with greater awareness related to lower QoL. These two domains reflect the person’s evaluation of day-to-day function, changes in which may have a particular significance in early-stage dementia. No association with QoL-AD was found for the Global Interview Rating, a global rating of awareness based on an extensive interview, or for awareness measures based on memory performance and on discrepancies in caregiver and person with dementia reports of social and emotional function. The awareness measure for evaluative judgements of functional abilities was included in the final model, with a borderline significant beta coefficient. Our data clearly indicates that awareness plays, if anything, a minor role in self-reports of QoL-AD in people with early stage dementia. As indicated above, the role of mood and self-concept in mediating the relationship between awareness and quality of life is revealed by our data.

Our findings on the Global Interview Rating are consistent with the only previous study that has used the QoL-AD, where a global rating of awareness or insight was not related to QoL scores [22]. It is also consistent with the conclusion from a further study [23] in relation to a sub-sample with mild impairment (MMSE of 21 and above) that simple self- and researcher-ratings of insight are not related to QoL in bivariate analyses (although they reported some association in a multivariate model with cognitive function (ADAS-Cog) also included). Our sample mainly comprises people with MMSE scores of 21 and over; 11 scored 18–20, but excluding these does not change our findings at all.

Although the current study has a good sample size, and has used a wider variety of potential predictors, including more refined measures of awareness, than previous studies, it is limited as a cross-sectional study, and in terms of the relative homogeneity of the population studied. Further work will explore changes in QoL over time, in relation to these potential predictors.

## Conclusions

This study indicates that QoL ratings by people with early-stage dementia are influenced by a complex variety of factors, and are not determined by one single aspect of the person’s life. They do reflect the person’s social climate, mood and self-concept, but, when considered alongside other variables, the influence of the trait of conscientiousness and of the person’s awareness of abilities and impairments (however this is assessed and measured), is relatively slight. This should give confidence to researchers and practitioners in continuing to use the QoL measures now available for people with early-stage dementia, and to take very seriously the voice and perspective of people with dementia regarding their situation and circumstances. Interventions aimed at improving mood, relationships and strengthening the self-concept may prove to be valuable in improving and maintaining quality of life in people with dementia.

## Competing interests

The authors declare that they have no competing interests.

## Authors’ contributions

LC was the Principal Investigator for the study. LC, RTW, RM, IM and IR conceived and designed the study. SMN, AM and JR contributed to the acquisition of data. CJW and RTW conducted the analyses and RTW drafted the manuscript. All authors contributed to the interpretation of data, commented on drafts of the manuscript and read and approved the final manuscript.

## Supplementary Material

Additional file 1 Word document comprising a Table of studies that have reported on factors predicting self-report QoL-AD scores in people with dementia. Click here for file
