# Supplementary material for: What contributes to a good quality of life in early dementia? awareness and the QoL-AD: a cross-sectional study
Source: Health Qual Life Outcomes. 2014 Jun 11;12:94. doi: 10.1186/1477-7525-12-94 (PMC4061777; doi:10.1186/1477-7525-12-94)
Supplement: Additional file 1 — Word document comprising a Table of studies that have reported on factors predicting self-report QoL-AD scores in people with dementia. [file 1477-7525-12-94-S1.docx]

**Additional File 1**

**Table of studies using self-reported QoL-AD in people with dementia**

| **Study** | **Location & sample size** | **Predictors (statistically significant relationship)** | **Total adjusted R2** | **Non-predictors** | **Mean MMSE (sd)** |
| --- | --- | --- | --- | --- | --- |
| Logsdon et al 2002 [5] | N=177 probable or possible AD, community sample  (155 able to complete QoL-AD) | Depression:  GDS (r=-0.51)  RMBPC Depression (r=-0.22)  Pleasant events scale (r=0.30)  ADL (r=-0.31)  SF-36 physical function (r=0.22)  SCB objective burden (r=-0.21)  SCB subjective burden (r=-0.19) | N/A | MMSE (r=0.12)  IADL (r=-0.07)  RMBPC Memory  RMBPC Disruption | 16.4 (7.3) |
| Thorgrimsen et al 2003 [29] | N=201  Care homes & day care | Depression :  Cornell Scale for Depression in Dementia (r=-0.20) | N/A | MMSE | 14.4 (3.8) |
| Shin, 2006 [7] | N=24, dementia  Community sample | NPI Total (r=-0.56)  NPI depression/dysphoria (r=-0.45)  NPI anxiety (r=-0.49)  NPI disinhibition (r=-0.42) | N/A | MMSE (r=0.097) | 16.5 (5.1) |
| Matsuia et al 2006 [10] | N=140 mild and moderate AD  Community resident | *Mild only:*  MMSE  *Mild & Moderate*  NPI: Mood factor  *Moderate only:*  NPI: Psychosis factor  (NPI = Frequency x severity) | N/A | *Mild:*  Age, gender, ADL, NPI psychosis, Short Memory Questionnaire (SMQ)  *Moderate:*  MMSE, ADL, SMQ, Age. Gender | N=88: 21 and above  N=52: 10-20  Mean 20.3 (4.2) |
| Vogel et al 2006 [22] | N=48 people with early AD  Community sample | No significant correlations identified | N/A | MMSE (r=-0.13)  Depression (GDS) (r=-0.22)  Frontal Behavioural Inventory (r=-0.10)  Cued recall test (r=-0.19) | 24.9 (2.3) |
| Hoe et al 2006 [8] | Total sample N=238 people with dementia  N=119 had self- and staff-completed QoL-AD  Care homes | Depression (Cornell) β=-0.40  Anxiety (RAID)β= -0.32 | 0.28 | MMSE  CAPE-BRS  Challenging Behaviour Scale  Barthel ADL  Clinical Dementia Rating  Met and unmet need (CANE) | Not provided for sample completing QoL-AD  (186 of total sample completed MMSE – mean 8.7 (7.8)) |
| Menne et al 2009 [9] | N=211  Community sample | African-American β=-0.19  Have spouse caregiver β=0.21  Depression (CES-D) β=-0.33  More involved in daily decision making β=0.19  Less negative strain in relationship with caregiver (rated by person with dementia) β=-0.11 | 0.38 | MMSE β=0.05  Age β=-0.07  Gender β=0.06  Time since diagnosis β=-0.03 | 22.01 (4.67) |
| Beer et al 2010 [12] | N=226  Resident in care facilities | Documented restraint β=-0.23 Reported pain β=-0.25  NPI-NH neuropsychiatric symptoms β=-0.16 | 0.13 | MMSE  Age  Gender | Median 17 |
| Naglie et al 2011 [11] | N= 370  Community sample probable AD  MMSE>10 | Depression (GDS-30) β=--2.83  NPI neuropsychiatric symptoms β=-0.80  (NPI – frequency x severity) | 0.42 | MMSE  ADAS-Cog  Disability Assessment for Dementia (Function)  Age, Gender. Education | 22.3 (4.3) |

*Abbreviations:*

ADAS-Cog – Alzheimer’s Disease Assessment Scale - Cognitive

ADL – Activities of Daily Living

CANE – Camberwell Assessment of Need for the Elderly

CAPE BRS – Clifton Assessment Procedures for the Elderly – Behaviour Rating Scale

CES-D – Center for Epidemiologic Studies - Depression scale

GDS – Geriatric Depression Scale

IADL – Instrumental Activities of Daily Living

MMSE – Mini-Mental State Examination

NPI – Neuropsychiatric Interview;

NPI-NH – Neuropsychiatric Interview – nursing home version

RAID – Rating of Anxiety in Dementia

RMBPC - Revised Memory and Behavior Problems Checklist

SCB - Screen for Caregiver Burden

SF-36 - Medical Outcomes Studies (36 item short-form measure)

SMQ – Short Memory Questionnaire
